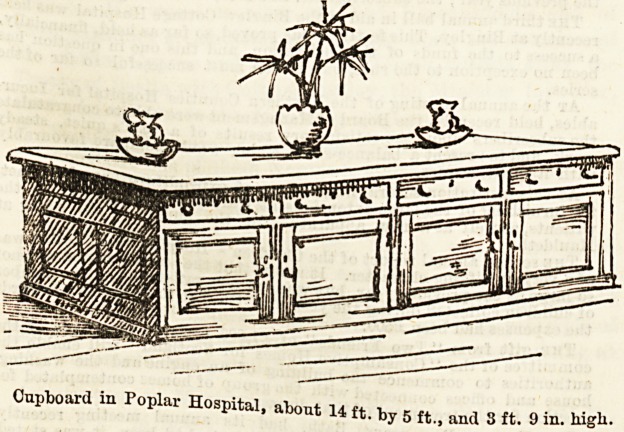# Appliances at Poplar

**Published:** 1894-01-27

**Authors:** 


### PRACTICAL DEPARTMENTS.

APPLIANCES AT POPLAR (*continued*).

Lockers.

In discussing the merits of the various forms of patients' lockers, we have before noticed the specially convenient feature which is noticeable in the sketch here shown. The lockers in the wards of the Poplar Hospital for Accidents are all made on this plan, and those who use them testify to their convenience.

Locker in Poplar Hospital, about 3 ft. 6 in. high.

It will be seen that the side next to the bed is altogether open, thus allowing each patient's special possessions to be within their easy reach. The lockers are 3 ft. 6 in. in height, bringing the second shelf on a comfortable level for the occupant of the bed. The tops are of marble. They are fairly capacious cupboards, quite large enough for their purpose, and are nicely finished. Like the cupboards, they are made in varnished wood, always clean-looking. The only possible fault to be found is that of their having been originally made minus air spaces, but this has been, or will be, put right by the drilling of a few holes in the back of each division, a simple and perfectly effectual method of ventilation which will put the lockers beyond criticism.

Ward Cupboards.

Much care and thought has been expended on all details of furniture and fittings by the Poplar Hospital authorities, and the cupboards are excellent of their kind. Our sketch shows the large store-cupboards occupying the centre of each of the

Cupboard in Poplar Hospital, about 14 ft. by 5 ft., and 3 ft. 9 in. high.

three wards, which at present form all the available part of the Accident Hospital. They are of noble proportions, and all manner of stores, linen, bandages, and other ward supplies are housed in their capacious depths.

14 by 5 ft. in length and width and about 3 ft. 9 in. in height, plenty of room is given for drawers in addition to the roomy cupboards, glimpses within which reveal shelves well furnished with the usual ward paraphernalia.

The cupboards are made of varnished wood, the tops being of scrubbed deal. For some reasons marble tops might be found preferable, but the plain wood has its advantages, and the general appearance leaves nothing to be desired.

Dainty little linen mats placed under the washhand basins, and pots of palms and ferns, of which there is no lack at the Poplar Hospital, add much to the attractive effect. At the ward's lower end are marble topped cupboards, of generous size, devoted to medicines and medical appliances of various kinds.

Outside the wards are the splint cupboards, well supplied with beautifully padded splints, "nearly all," said the nurse who kindly threw open her stores for our inspection, "made by a patient," whose deft-fingered work did him immense credit, and whose help must have been valued indeed by the busy nurses.

There is not much scope for the critic in discussing the comparative merits of these practical ward accessories, for each is good, and well worthy of the attention of those desirous of gaining hints on the best methods of furnishing hospital wards.

## Figures and Tables

**Figure f1:**
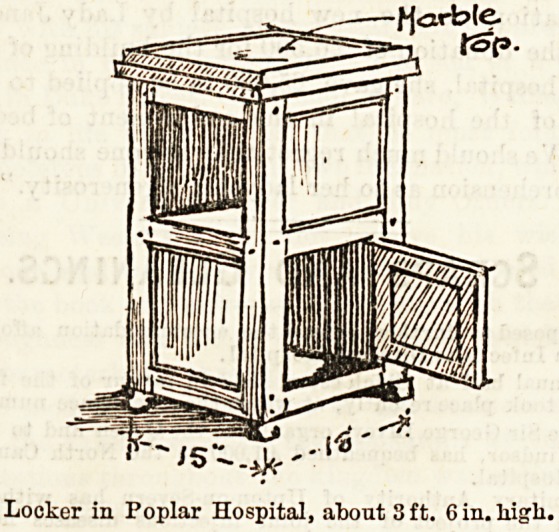


**Figure f2:**